# Aperture Proximity Effects in High Heat Flux Sensors Calibration

**DOI:** 10.6028/jres.103.041

**Published:** 1998-12-01

**Authors:** A. V. Murthy, B. K. Tsai, R. D. Saunders

**Affiliations:** Aero-Tech, Inc., Hampton, VA 23666; National Institute of Standards and Technology, Gaithersburg, MD 20899-0001

**Keywords:** calibration, heat flux, radiation, sensors

## Abstract

In the transfer calibration of heat flux sensors, a correction for the irradiance distribution across the sensing area may be required when the sensing areas of the reference and test sensors are different. A method to calculate this correction using well-known equations for the configuration factors is presented. Also, estimates of the correction for test conditions corresponding to the transfer calibration technique in use at the National Institute of Standards and Technology are given.

## 1. Introduction

The calibration of high heat flux sensors using radiant blackbody sources requires positioning of the sensors close to the aperture of the radiant source [1]. Hence the heat flux measured or calculated at the sensor location is sensitive to uncertainties in location of effective aperture, positioning accuracy, and finite size of the sensor. In particular, when transferring the calibration from a radiometer whose aperture is larger than the sensitive area of the sensor to be calibrated, the averaging areas for the radiometer and the sensor will be different. This does not present a problem when the sensors are located far away from the radiant source, since the heat flux distribution is uniform over the sensing area. However, for sensor locations close to the aperture, it is necessary to ascertain that the effect due to non-uniform distribution of the heat flux is small or a correction is applied, if necessary. These effects are dependent only on the geometry of the calibration setup and can be calculated using well-known equations for the configuration factor [2]. This paper presents the relevant calculations and gives estimates of the corrections representative of the test conditions in the transfer calibration technique at the National Institute of Standards and Technology [1].

## 2. Theory

**Fig. 1:**
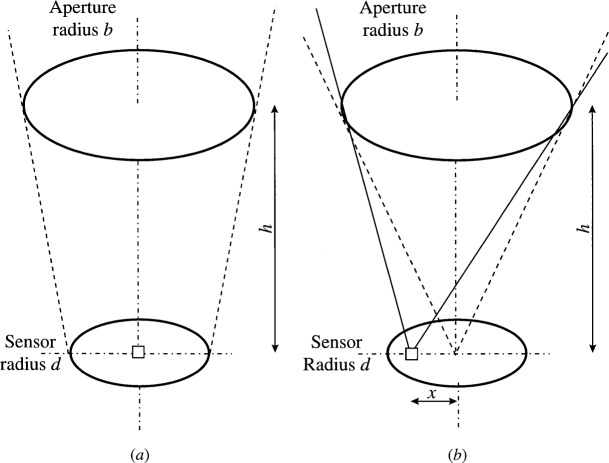
Configuration factor between two disks: (a) average, (b) local.

The radiation exchange between two surfaces is determined by the geometric view factor. For exchange between two circular disks (Fig. 1a), corresponding to the transfer calibration setup at NIST, the average configuration factor *C*_fa_ is given by

$$C_{\mathrm{fa}}=\left[\frac{(1+B^{2}+C^{2})-{\left[{\left(1+B^{2}+C^{2}\right)}^{2}-4B^{2}C^{2}\right]}^{1/2}}{2B^{2}}\right],$$ (1)

where *B* = *b*/*h*, *C* = *d*/*h*, and *b* is the radius of the radiating aperture, *d* is the radius of the sensor and *h* is the distance between the radiating aperture and the sensor. By differentiating Eq. (1) with respect to distance *h*, we obtain an expression for relative change in the value of the configuration factor for small variations in the distance caused by uncertainties in positioning the sensor or the exact location of the aperture. This variation is represented by

$$\frac{dC_{\mathrm{fa}}}{C_{\mathrm{fa}}}=-2{\left[{\left(1+B^{2}+C^{2}\right)}^{2}-4B^{2}C^{2}\right]}^{-1/2}\left(\frac{dh}{h}\right).$$ (2)

When the sensor size is finite, the viewing angle varies across the sensor surface (Fig. 1b). Hence the local configuration factor varies from the center to the outer edge of the sensor. The local value of the configuration factor *C*_fa_ at a distance *x* from the center is given by the expression

$$C_{fx}=\left(\frac{1}{2}\right)\left[1-\frac{\left(1+D^{2}-B^{2}\right)}{{\left[D^{4}+2D^{2}(1-B^{2})+{\left(1+B^{2}\right)}^{2}\right]}^{1/2}}\right],$$ (3)

where *D* = *x*/*h*, *B* = *b*/*h*, and *x* is the distance from the center of the sensor. At the center (*x* = 0), and Eq. (2) simplifies to

$$C_{f0}=\left[\frac{B^{2}}{\left(1+B^{2}\right)}\right].$$ (4)

## 3. Results and Discussion

Numerical values have been calculated for the aperture and sensor sizes corresponding to the testing geometry in the transfer calibration setup. For heat flux sensors calibrated, or likely to be calibrated in the future, the sensitive area of the sensor will be much less than the diameter of the radiating aperture. For the transfer standard radiometer currently in use, the aperture area is 1.0 cm^2^. For calibrations using the 25 mm Variable Temperature Blackbody (VTBB), the ratio of the radiometer and blackbody aperture diameters will be 0.45. Hence the results of the calculations are presented for the range of diameter ratios 0.1, 0.3, and 0.5.

**Fig. 2:**
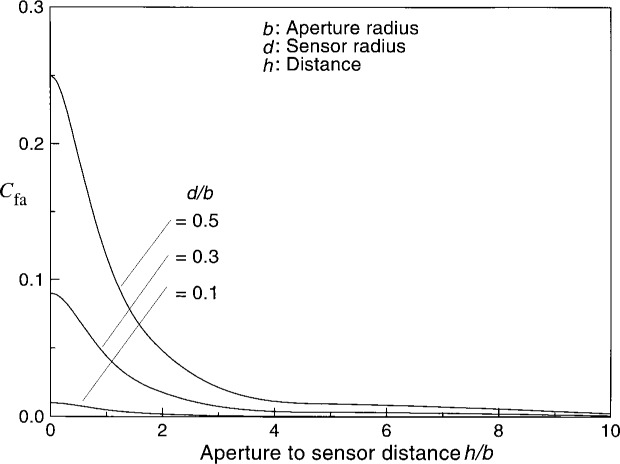
Configuration factor between two circular disks.

**Fig. 3:**
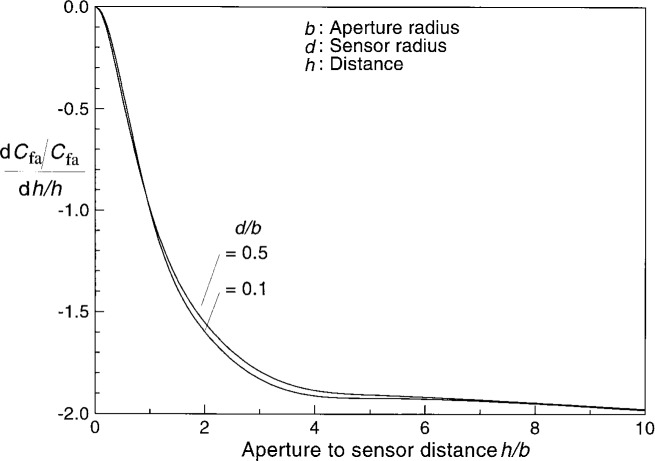
Relative change in configuration factor between two disks for small variation in the separation distance.

Figure 2 shows the typical variation of average configuration factor *C*_fa_ with distance. When the sensor is at the radiating aperture location, the value corresponds to the ratio of the areas. Away from the aperture, the configuration factor drops rapidly to the location where *h*/*b* ≈ 2. Farther away, the decrease is gradual. From Fig. 3, it may be seen that the rate of change of configuration factor with distance is almost independent of the radius of the receiving disk (sensor). The rate of change is zero when the sensor is at the aperture location and decreases to about −1.9 when it is at a distance of twice the aperture diameter. Farther away, it gradually approaches the asymptotic value of −2. This means that the fractional change in configuration factor is about twice the uncertainty in the location of the sensor. However, the relative uncertainty of the sensor position is small when the sensor is located farther from the aperture. When the sensor is closer to the aperture, for a given positioning accuracy Δ*h*, the relative uncertainty Δ*h*/*h* will be much higher. In addition, cooling around the aperture can make it difficult to define the exact location of the effective radiating aperture. The cooling effect is difficult to estimate and can be a major source of uncertainty when the sensor is located close to the aperture to achieve high heat flux levels.

**Fig. 4:**
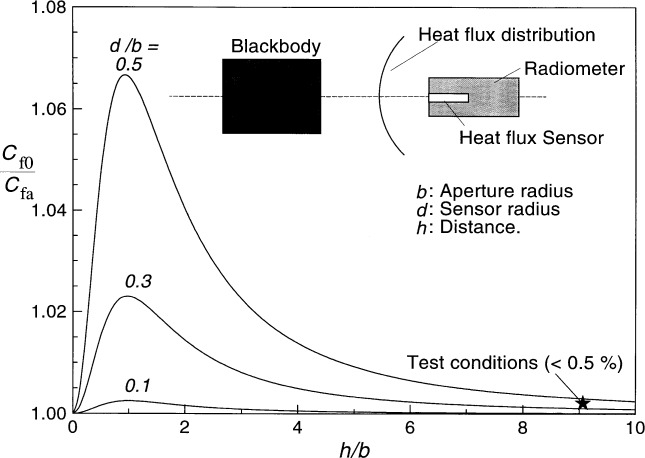
Correction factor for the reference radiometer reading.

Another effect to be examined in the transfer calibration is the averaging area of the sensor and the transfer standard. Downstream of the radiating aperture, the heat flux distribution has a peak at the center and decreases away from the center. The sensitive area of the heat flux sensor is generally small, and responds to the peak of the distribution. However, the aperture size of the transfer standard cavity-type electrical substitution radiometer is much larger. Hence the response of the radiometer will be proportional to the average flux captured from the distribution. Due to this averaging effect, a correction to the radiometer measurement may be necessary in some cases to determine the peak value of the distribution from the average reading. This correction can be estimated by considering the variation of the configuration factor across the radiometer aperture, and plotting the ratio at *x* = 0, to the average value (*C*_fo_/*C*_fa_). Figure 4 shows this correction for different sensor dimensions versus the location of the sensor. The correction factor is unity when the sensor is located at the aperture plane because of the uniform distribution. The correction factor peaks approximately at a distance equal to the radius of the aperture, and decreases asymptotically to unity at large distances. The correction is a strong function of the ratio of the sensor to the aperture radii. For the VTBB test conditions, this ratio is about 0.44, and the correction is less than 0.5 % of the measured radiometer reading. The correction increases rapidly when moving closer to the aperture.

## 4. Conclusions

An analysis of the geometrical effects of calibrating heat flux sensors in a blackbody environment is presented. Factors such as average configuration factor *C*_fa_, its sensitivity with separation distance *h*, and a correction for non-uniform distribution of the irradiance at the sensor location are considered. For typical NIST transfer calibration configurations, the correction for measured sensor responsivity due to non-uniform distribution is less than 0.5 %, as long as the test sensor is separated from the blackbody aperture by at least four aperture diameters. The corresponding positioning and configuration factor uncertainties will also remain small for a wide range of sensor to blackbody apertures radii.

## References

[1] Murthy AV, Tsai BK, Gibson CE (1997). Calibration of High Heat Flux Sensors at NIST. J Res Natl Inst Stand Technol.

[2] Rall LD, Hornbakeer RD Radiometer View Angle, Its Meaning with Respect to Instrument Applications and Specifications, TR-193.

